# The Contribution of Scalded and Scalded-Fermented Rye Wholemeal Flour to Quality Parameters and Acrylamide Formation in Semi-Wheat-Rye Bread

**DOI:** 10.3390/foods12050937

**Published:** 2023-02-22

**Authors:** Dovile Klupsaite, Vytaute Starkute, Egle Zokaityte, Darius Cernauskas, Ernestas Mockus, Evaldas Kentra, Rugilė Sliazaite, Gabriele Abramaviciute, Paulina Sakaite, Vitalija Komarova, Ieva Tatarunaite, Sandra Radziune, Paulina Gliaubiciute, Monika Zimkaite, Julius Kunce, Sarune Avizienyte, Milena Povilaityte, Kotryna Sokolova, João Miguel Rocha, Fatih Özogul, Elena Bartkiene

**Affiliations:** 1 Institute of Animal Rearing Technologies, Faculty of Animal Sciences, Lithuanian University of Health Sciences, Tilzes Str. 18, LT-47181 Kaunas, Lithuania; 2 Department of Food Safety and Quality, Veterinary Academy, Lithuanian University of Health Sciences, Tilzes Str. 18, LT-47181 Kaunas, Lithuania; 3 Food Institute, Kaunas University of Technology, Radvilenu Road 19, LT-50254 Kaunas, Lithuania; 4 Laboratory for Process Engineering, Environment, Biotechnology and Energy (LEPABE), Faculty of Engineering, University of Porto (FEUP), Rua Dr. Roberto Frias, 4200-465 Porto, Portugal; 5 Associate Laboratory in Chemical Engineering (ALiCE), Faculty of Engineering, University of Porto (FEUP), Rua Dr. Roberto Frias, 4200-465 Porto, Portugal; 6 Department of Seafood Processing Technology, Faculty of Fisheries, Cukurova University, Balcali, Adana 01330, Turkey; 7 Biotechnology Research and Application Center, Cukurova University, Balcali, Adana 01330, Turkey

**Keywords:** bread, scalding, fermentation, acrylamide, lactic acid bacteria

## Abstract

The aim of this study was to evaluate the influence of scalded (Sc) and scalded-fermented (FSc) (with *Lactiplantibacillus paracasei* No. 244 strain) rye wholemeal flour on the quality parameters and acrylamide formation in semi-wheat-rye bread. To that purpose, 5, 10 and 15% of Sc and FSc were used for bread production. Results showed that scalding increased fructose, glucose and maltose content in rye wholemeal. Lower concentrations of free amino acids were found in Sc when compared with rye wholemeal, but fermentation of Sc increased the concentrations of some amino acids (on average by 1.51 times), including gamma aminobutyric acid (GABA, by 1.47 times). Addition of Sc and FSc had a significant influence (*p* ≤ 0.05) on bread shape coefficient, mass loss after baking and most bread colour coordinates. Most of the breads with Sc or FSc showed lower hardness after 72 h of storage compared with the control (i.e., without Sc or FSc). FSc improved bread colour and flavour, as well as overall acceptability. Breads with 5 and 10% of Sc had a similar level of acrylamide to the control, while its level in breads with FSc was higher (on average, 236.3 µg/kg). Finally, different types and amounts of scald had varying effects on the quality of the semi-wheat-rye bread. FSc delayed staling and improved sensory properties and acceptability, as well as the GABA level of wheat-rye bread, while the same level of acrylamide as was seen in control bread could be reached when using between 5 and 10% of scalded rye wholemeal flour.

## 1. Introduction

Wheat bread is the most popular bread in many countries and accepted as a very convenient form of high energy food with good digestibility [1]. However, semi-wheat-rye bread takes a higher position in the East European market; it is also considered to be better balanced, because of the presence of various cereal varieties with differing compositions [2]. In the production of semi-wheat-rye bread, wheat flour has a very important technological function because of its gluten network, which gives elasticity to the wheat–rye dough [3]. This characteristic leads to better gas retention and improves the gas extension in the dough, leading to a higher porosity and specific volume of the bread [4].

Owing to consumer interest in healthy nutrition and taking into consideration that bread is a very popular product consumed on a daily basis, bakeries are always eager to produce healthier products [5]. One of the steps to improve bread properties is to eliminate added sugar (saccharose) from the main bread formula [6].

However, the sensory properties of bread are very important characteristics of bread choice, and most consumers prefer a sweeter tasting product [7]. The influence of saccharose consumption on public health continues to be a controversial topic but it is known that the consumption of added sugar is associated with the risk of developing a range of chronic diseases [8,9,10,11,12]. To avoid numerous health problems associated with added sugar consumption, the World Health Organization (WHO) published guidelines in 2015 that recommended reducing the intake of “free sugars” to <10% of calories per day [13].

The use of scalded flour (Sc) can be an attractive technology to increase the sweet taste of bread without addition of saccharose, and it has indeed been reported that scalded flour has a positive influence on glycaemic index [14]. This technology is very common in Eastern Europe because it can ensure the good characteristics and sweeter taste of cereal products without any additional sugar. In addition, scalded flour is a good substrate for the growth of lactic acid bacteria (LAB), which are usually used in bread production as a microbial starter culture for sourdough fermentation. These suitable characteristics for LAB growth and multiplication are associated with the resulting hydrolysis of the flour starch and production of fermentable sugars (usually monosaccharides) that are easily available for LAB consumption and their metabolic conversion of monosaccharides to organic acids and other important metabolites. Additionally, fermentation of scalded flour leads to the desirable sweet–sour taste of bread, which is generally preferred by consumers. Furthermore, the combination of these two steps—i.e., scalding and fermentation—can lead to the extension of bread shelf-life as well as acrylamide reduction, owing to the degradation and metabolism of acrylamide precursors in baking dough.

The European Food Safety Authority (EFSA) scientific opinion on acrylamide in foods concluded that dietary exposure to acrylamide potentially increases the risk of developing cancer for consumers in all age groups; thus, the food industry should reduce acrylamide concentration in foods [15]. Acrylamide is formed in food products during their thermal treatment at temperatures > 120 °C, especially during the Maillard reaction. Formation of acrylamide in bread depends on many factors, including temperature, processing, type of flour(s), recipe, etc. [16].

One of the possibilities for reducing acrylamide formation in bread is the use of sourdough technology because of the rapid pH drop and degradation of the main precursors of acrylamide in dough that can be achieved using selected LAB strains. However, studies on the influence of scalded flour fermented with *Lactiplantibacillus paracasei* on acrylamide formation in bread are limited. Moreover, it should be pointed out that the scalding technology increases acrylamide precursor (monosaccharides) formation in dough. For this reason, not only should consumer preference for a sweet-tasting product be considered, but also the safety parameters, i.e., acrylamide formation in bread prepared with scalded flour.

Our previous studies focused on acrylamide concentration reduction in cereal products (mostly in bread and biscuits) using selected LAB strains and their combinations, showed very promising results, albeit by selecting the most appropriate LAB strain for each product technology [17,18,19,20,21,22,23,24,25,26]. These previous findings were explained by the different composition of the fermentable substrate, which led to different metabolic activities of the employed LAB, as well as differing efficiencies in the reduction of the acrylamide precursors in dough and, subsequently, acrylamide concentration in the end product.

Despite significant progress being made in this field since our research was published, in general, studies about the influence of scalded flour and scalded flour fermented with *Lactiplantibacillus paracasei* (FSc) on acrylamide formation in semi-wheat-rye bread are still scarce.

The aim of this study was to evaluate the influence of scalded and scalded-fermented (with *Lactiplantibacillus paracasei* No. 244 strain) rye wholemeal flour on the quality parameters and acrylamide formation in semi-wheat-rye bread. For this purpose, different quantities of scalded (Sc) and scalded-fermented (FSc) rye wholemeal flour were tested for semi-wheat rye bread (W-R) preparation (5, 10 and 15%). In addition, Sc and FSc parameters (pH, total titratable acidity (TTA), colour characteristics, hardness, sugars (fructose, glucose, sucrose and maltose) concentration and amino acid profile), as well as W-R quality and safety characteristics (specific volume, shape coefficient, mass loss after baking, crust and crumb colour coordinates, sensory characteristics, overall acceptability, and acrylamide concentration) were analysed.

## 2. Materials and Methods

### 2.1. Materials Used for Bread Preparation

Wheat flour (type 550D, gluten 26%, carbohydrate content 68%, fibre content 3.9%, protein content 11.9%, fat content 1.7% and ash 0.55–0.62%) and rye wholemeal flour (fat content 1.1%, carbohydrate content 62.2%, fibre content 16% and protein content 8.5%) obtained from ‘Malsena plius’ Ltd. mill (Panevezys, Lithuania) were used for the W-R preparation. The W-R samples were prepared without and with addition of Sc and FSc (5, 10 and 15%).

*Lactiplantibacillus paracasei* No. 244 strain showing versatile carbohydrate metabolism and tolerance to acidic conditions [27] was used for FSc preparation. Strain No. 244 was stored at −80 °C in a Microbank system (PRO-LAB DIAGNOSTICS) and propagated in a DeMan, Rogosa and Sharpe (MRS) broth (CM 0359; Oxoid Ltd., Hampshire, UK) at 30 °C for 48 h. This LAB strain was previously newly isolated and identified from a spontaneous rye sourdough, which is traditionally used in rye bread production [27]. The characteristics of the *Lp. paracasei* No. 244 strain are given in Table 1.

### 2.2. Scald Preparation and Fermentation

The Sc was prepared by using 1000 g of rye wholemeal flour mixed with 1000 mL of hot water (95 °C). The scalding process was carried out at 30 °C for 2 h. For Sc fermentation *Lp. paracasei* No. 244 strain was used. The *Lp. paracasei* No. 244 cell suspension (5 mL), containing about 8.9 log_10_ CFU/mL, was added into the Sc (cooled to 30 °C), followed by fermentation for 24 h at 30 °C. Prepared Sc and FSc samples were applied for W-R preparation by using 5, 10 and 15% (% of the total flour content).

### 2.3. Breadmaking

**Figure 1:**
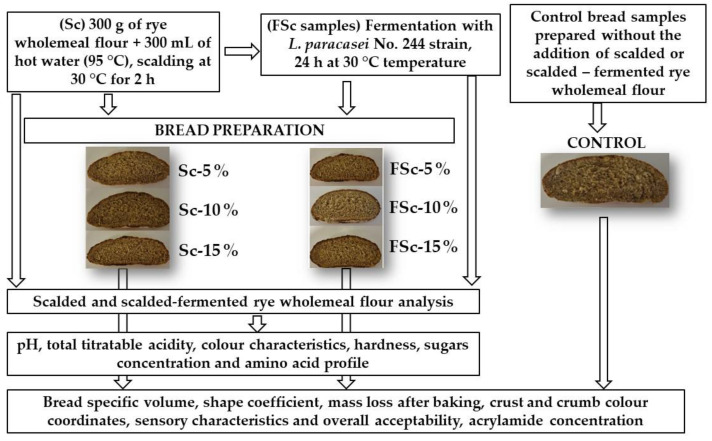
Schematic representation of the experimental design.

The W-R formula consisted of 0.5 kg wheat flour, 0.5 kg rye flour, 1.5% salt, 3% instant yeast and 1000 mL water (control bread). Control W-R samples were prepared without the addition of Sc or FSc. The tested W-R groups were prepared by addition of 5, 10 and 15% Sc or FSc to the main recipe. In total, seven groups of baking dough and respective W-R samples were prepared and tested. The dough was mixed for 3 min at a low speed, then for 7 min at a high-speed regime in a dough mixer (KitchenAid Artisan, OH, USA). Then, the dough was left at 22 ± 2 °C for 15 min relaxation. Subsequently, the dough was shaped into 425 g loaves, formed and proved at 30 ± 2 °C and 80% relative humidity for 60 min. The bread was baked in a deck oven (EKA, Borgoricco, PD, Italy) at 220 °C for 25 min. The schematic representation of the experimental design is shown in Figure 1.

### 2.4. Evaluation of Non-Treated, Scalded (Sc) and Scalded-Fermented (FSc) Rye Wholemeal Flour Parameters

The Sc and FSc samples (after 0 (FSc-0h) and 24 (FSc-24h) h of fermentation) were analysed to evaluate their pH, TTA, colour characteristics, hardness, sugar (fructose, glucose, sucrose and maltose) concentration and amino acid profile.

The pH values of Sc and FSc were measured and recorded with a pH electrode (PP-15; Sartorius, Göttingen, Germany). For pH analysis, the electrode was immersed directly into the Sc or FSc sample.

The TTA was determined for a 10 g sample of Sc or FSc homogenized with 90 mL of distilled water and expressed as mL of 0.1 mol/L NaOH required to achieve a pH of 8.2.

Colour parameters were evaluated using a CIE L*a*b* system (CromaMeter CR-400, Konica Minolta, Japan) [28].

The hardness of Sc and FSc samples was measured as the energy required for sample deformation (CT3 Texture Analyzer, Brookfield, Middleboro, USA), viz.: 50 g of a Sc or FSc sample was placed in a Petri dish and compressed to 10% of its original height at a crosshead speed of 0.5 mm/s; the resulting peak energy of compression was reported as Sc or FSc sample hardness.

To determine the sugar concentration, 1–2 g of sample was diluted in 60 mL of distilled/de-ionized water, heated to 60 °C in a water bath for 15 min, clarified with 2.5 mL Carrez I (85 mM K_4_[Fe(CN)_6_] × 3H_2_O) and 2.5 mL Carrez II (250 mM ZnSO_4_ × 7H_2_O) solutions, and made up to 100 mL with distilled/de-ionized water. After 15 min, the samples were filtered through a filter paper and a 0.22 μm nylon syringe filter before further analysis. A 2 mg/mL standard solution of a sugar mixture was prepared by dissolving 0.2 g of each of fructose, glucose, sucrose and maltose (Sigma-Aldrich, Hamburg, Germany) in 100 mL of distilled/de-ionized water. Chromatographic conditions were as follows: the eluent was a mixture of 75 parts by volume of acetonitrile and 25 parts by volume of water, the flow-rate was 1.2 mL/min, 20 μL was injected. A YMC-Pack Polyamine II 250 × 4.6 mm, 5 μm (YMC Co., Ltd., Tokyo, Japan) column was used. The column temperature was set at 28 °C. The detection was performed using an Evaporative Light Scattering Detector (ELSD) LTII (Shimadzu Corp., Kyoto, Japan).

For free amino acids and gamma aminobutyric acid (GABA) analysis, the homogenized sample (~100 mg) was weighed into a 1.5 mL tube and analytes were extracted with 1 mL of aqueous 0.1 M HCl solution by shaking for 1 h. The resultant mixture was centrifuged at 12,000 rpm for 5 min. For derivatization, 50 µL of the resultant supernatant was mixed with 100 µL of 100 mg/L diaminoheptane (as an internal standard) and diluted to 500 µL with 0.1 M HCl solution. The resultant mixture was alkalized by addition of 40 µL of 2 M NaOH and 70 µL of the saturated NaHCO_3_ solution. Derivatization was performed by adding 1 mL of 10 mg/mL dansyl chloride solution in acetonitrile and incubating the resulting mixture at 60 °C for 30 min. The reaction mixture was quenched using 50 µL of 25% ammonia solution and filtered through a 0.22 µm membrane filter into the auto-sampler vial. The concentration of analytes was determined using a Varian ProStar HPLC system (two ProStar 210 pumps and a ProStar 410 autosampler; Varian Corp., Palo Alto, CA, USA) and a Thermo Scientific LCQ Fleet Ion trap mass detector (Thermo Fisher Scientific, San Jose, CA USA). For analyte detection, the mass spectrometer was operated in positive-ionization single-ion monitoring mode for specific ions corresponding to derivatized analytes. The analyte concentration was determined from a calibration curve, which was obtained by derivatizing the analytes at different concentrations. For the separation of derivatives, a Discovery**^®^** HS C18 column (150 × 4.6 mm, 5 µm; SupelcoTM Analytical, Bellefonte, PA, USA) was used. Mobile phase A was 0.1% formic acid in 5% aqueous acetonitrile, and phase B was 0.1% in acetonitrile (% in volume). A flow-rate of 0.3 mL/min was used for the analysis. The injection volume was 10 µL. The analytical gradient was as follows: 0 to 10 min (linear gradient) 15 to 60% B, 10 to 40 min (linear gradient) 60 to 95% B, 40 to 48 min 95 B, followed by re-equilibration of the column for 10 min with 15% B (increased to 0.6 mL/min flowrate). The limit of quantification (according to the lowest concentration used for calibration) was 0.02 µmol/g.

### 2.5. Evaluation of Bread Quality

After 12 h of cooling at 22 ± 2 °C, W-R samples were subjected to analysis of specific volume, crumb porosity, shape coefficient, mass loss after baking, crust and crumb colour coordinates, sensory characteristics, overall acceptability and acrylamide concentration.

Bread volume was established by the AACC method [29], and the specific volume was calculated as the ratio of volume to weight. The bread shape coefficient was calculated as the ratio of bread slice width to height (in mm). Mass loss after baking was calculated as a percentage by measuring loaf dough mass before baking and after baking. Crust and crumb colour parameters were evaluated using a CIE L*a*b* system (CromaMeter CR-400, Konica Minolta, Tokyo, Japan) [28]. Bread crumb hardness was determined as the energy required for sample deformation (CT3 Texture Analyzer, Brookfield, Middleboro, USA): bread slices of 2 cm thickness were compressed to 10% of their original height at a crosshead speed of 0.5 mm/s; the resulting peak energy of compression was reported as crumb hardness. Three replicates from three different sets of baking were analysed and averaged.

Sensory characteristics and overall acceptability of breads was carried out by 10 trained judges according to the ISO method [30] using a 140 mm hedonic line scale ranging from 140 (like extremely) to 0 (dislike extremely).

### 2.6. Determination of Acrylamide in Bread

The acrylamide concentration was determined according to the method of Zhang et al. [31] with modification. The bread samples were homogenized in a blender (Ika A10, Staufen, Germany). Two grams of sample were weighed in a 50 mL centrifuge tube and diluted with 20 mL of distilled/de-ionized water. The sampling tube was briefly vortexed (ZX3 Advanced VELP, Usmate (MB), Italy) to mix the contents for 10 min. The sample tube was centrifuged at 4000 rpm for 10 min with a centrifuge (Hermle Z 306, HERMLE Labortechnik GmbH, Wehingen Germany). Next, 10 mL samples of the clarified aqueous layer solution were transferred to 15 mL centrifuge tubes and clarified with 100 µL of Carrez I (85 mM K_4_[Fe(CN)_6_] × 3H_2_O) and 100 µL of Carrez II (250 mM ZnSO_4_ × 7H_2_O) solutions. The sample tubes were then centrifuged at 4000 rpm for 10 min.

For the preparation of acrylamide standard solution (30.4 µg/L), 15.2 mg of acrylamide analytical standard (99.8% purity) was weighed and dissolved in a 1000 mL volumetric flask and diluted with de-ionized water. The obtained solution was diluted by pouring 2 mL of the obtained acrylamide solution into a 1000 mL measuring flask and diluted with de-ionized water.

Three millilitres of the sample supernatant (or standard solution) was derivatized in a glass sample tube by adding 1.5 g of potassium bromide (KBr), 1 mL of potassium bromate solution (0.1 M, KBrO_3_) and 0.3 mL of sulphuric acid solution (50%, H_2_SO_4_). The mixture was mixed in a shaker and kept for 2 h in a refrigerator (~4 °C). The derivative was neutralized by adding 250 µL of sodium thiosulphate solution (1 M, Na_2_S_2_O_3_ × 5H_2_O) until the orange colour disappeared. About 1.5 g of sodium chloride (NaCl) was added to the derivatization mixture and the mixture was extracted with ethyl acetate (CH_3_COOC_2_H_5_) (2 × 5 mL). The collected ethyl acetate was concentrated with a concentration system (Christ CT 02-50, Frankfurt, Germany) at a temperature of 40 °C and under reduced pressure. The solvent was evaporated and dissolved in 0.5 mL of ethyl acetate (for the standard, in a volume of 3 mL). Next, 100 mg of anhydrous sodium sulphate (Na_2_SO_4_) and 20 µL of triethylamine ((C_2_H_5_)_3_N) (20 µL of triethylamine in 0.5 mL of a concentrated derivatization solution) were added to the solution in a 15 mL centrifuge tube, mixed and centrifuged for 10 min (4000 rpm). The supernatant was analysed with a gas chromatograph-electron capture detector (GC–ECD).

A gas chromatograph (Shimadzu GC-17A, Tokyo, Japan) was equipped with an electron capture detector (ECD), an integrator to measure peak areas, and a thermostated column. The capillary column was a Rxi-5Sil MS (Restek, Germany): length 30 m; inner diameter 0.25 mm; stationary phase film thickness 0.25 µm. Working conditions were: injection volume 1 μL; column temperature gradient 70 °C (hold 1 min), 3 °C/min to 140 (hold 0.5 min), and 15 °C/min to 280 (hold 4 min). The mobile phase was nitrogen at 18.0 cm/sec flow rate, with a split of 3.0. The injector temperature was 250 °C, the detector temperature was 260 °C and the detector current was 2 nA.

### 2.7. Statistical Analysis

The results were expressed as mean values (for baking dough and bread samples n = 3, and for bread sensory characteristics and overall acceptability n = 10 trained panellists) ± standard error (SE). In order to evaluate the effects of different quantities of scalded non-fermented and fermented rye wholemeal flour on semi-wheat-rye bread quality parameters, data were analysed using a one-way ANOVA and Tukey-HSD as post-hoc tests (statistical program R 3.2.1). Additionally, Pearson correlations were calculated between various parameters, as well as between the dough and scald characteristics with acrylamide content. The results were recognized as statistically significant at *p* ≤ 0.05.

## 3. Results and Discussion

### 3.1. Parameters of Scalded (Sc) and Scalded-Fermented (FSc) Rye Wholemeal Flour

Acidity characteristics (pH and TTA), colour coordinates and hardness of Sc and FSc (after 0 and 24 h of fermentation) are shown in Table 2. When comparing the acidity parameters (pH and TTA) of the samples, the addition of pure LAB strains decreased pH and increased the TTA by 4.34 and 18.3%, respectively. After 24 h of fermentation, the pH of the samples was reduced to 4.57 and the TTA was increased to 2.53 °N. However, significant correlation between the pH and TTA of the samples was not found. The decrease in pH and increase in TTA in FSc samples are mainly related to organic acid production by lactic acid bacteria and their ability to acidify the fermentable substrates [32].

Regarding the colour coordinates of the samples, significantly higher values of redness (a*) (by 13.8%), and similar values of lightness (L*) and yellowness (b*) in FSc samples after 24 h of fermentation were found, when compared with Sc samples. Fermentation with LAB may induce the release of such pigments as anthocyanins and phenolic compounds, which are present in the pericarp, testa and aleurone layer of the rye grains [33,34,35]. This could cause the observed increased values of the a* coordinate in scalded-fermented rye flour.

The hardness of FSc samples was around 3.5 times lower in comparison with non-fermented-scalded rye flour. This may be explained by the proteolytic activity of the LAB strain and the acidity-elicited activation of proteolytic enzymes in flour [36]. The activity of the proteolytic enzymes induces the weakening of the gluten network structure and decreases the hardness of the fermented product [37].

The sugar content is depicted in Table 3. Fructose and glucose were absent in non-scalded rye wholemeal flour. However, the scalding process led to fructose and glucose formation (in the Sc sample, the fructose and glucose content was, on average, 0.880 and 1.25 g/100 g, respectively). Moreover, the scalding process increased maltose concentration by 6.14 times, in comparison with non-scalded flour. In both the Sc and FSc samples, sucrose was not detected; in addition, fermentation of the scalded flour significantly increased the glucose concentration by 13.2% in comparison with Sc. Fructose and maltose content in Sc and FSc remained similar, on average, 0.850 and 6.61 g/100, respectively. Due to the gelatinization of starch during scald production, amylases in flour tend to convert starch into maltodextrins and further into maltose and glucose [32]. This explains the results observed in our study, which are similar with those reported by Li et al. [38]. Despite the utilization of monosaccharides by LAB, most LAB strains, including *Lp. paracasei*, possess amylolytic activity and this could also cause the increase in mono- and disaccharides in fermented scald [39].

The content of free amino acids and GABA is given in Table 4. Concerning the free amino acid concentration, in the Sc and FSc samples the concentration was lower in most cases, in comparison with non-treated rye wholemeal flour: asparagine, on average, by 2.34 times; serine, on average, by 2.17 times; aspartic acid, on average, by 4.46 times; and proline, on average, by 2.31 times. In comparison, when considering arginine, glutamic acid, threonine, glycine and alanine concentrations, the highest content of these amino acids was found in non-treated flour in all cases. Moreover, when comparing Sc and FSc, the latter showed the higher content of these amino acids (on average, by 1.26, 1.14, 1.78, 1.66 and 1.71 times, respectively). An exception was observed with proline, the content of which in both the Sc and FSc samples was similar (on average, 0.968 µmol/g). The lower free amino acid content in Sc samples could be related to the protein denaturation or dilution effect [40]. Conversely, the higher content of some free amino acids in FSc may have occurred due to proteolytic activity in the LAB strain [41]. It has been reported that the content of such amino acids as tryptophan, glutamic acid, isoleucine, leucine and asparagine increased after fermentation of rye dough [42,43].

*Vis-à-vis* the gamma aminobutyric acid concentration in non-treated and scalded flour, significant differences were not observed, and GABA concentration was, on average, 0.469 µmol/g. However, in FSc samples GABA concentration was found to be 1.47 times higher, in comparison with non-treated flour and Sc samples. GABA possesses anticarcinogenic, antihypertensive, antidepressant and antidiabetic properties. It is a metabolic product of plants and microorganisms such as LAB; therefore, fermented foods are a potential source of this amino acid [44]. It has been reported that this health-promoting compound is found in rye malt sourdough after fermentation with *Limosilactobacillus reuteri* LTH5448 and LTH5795; wheat sourdough fermented with *Levilactobacillus brevis* CECT 8183; legume flour sourdough started with *Lev. brevis* AM7 and *Lactiplantibacillus plantarum* C48; and wheat, barley, chickpea, lentil and quinoa flour sourdoughs fermented with strains of *Lp. plantarum*, *Furfurilactobacillus rossiae* and *Fructilactobacillus sanfranciscens* [45,46]; however, no data on GABA concentration in Sc and FSc were found in the literature. The results obtained in our study show that the scalding process had no significant impact on the GABA content of rye flour, but fermentation of Sc with *Lactiplantibacillus paracasei* No. 244 strain increased GABA content by, on average, 1.47 times.

### 3.2. Bread Quality

The bread specific volume, porosity, shape coefficient, mass loss after baking, colour characteristics of the bread crust and crumb, as well as bread crumb images are given in Table 5. Observing the results of the specific volume of bread samples, no significant differences were detected and, on average, bread specific volume was 1.98 cm^3^/g. In comparison, for bread shape coefficient, samples prepared with 15% of Sc and FSc showed a lower shape coefficient in comparison with other bread groups (on average, 30.2% lower). The scald quantity used for bread preparation, and the scald fermentation and quantity interaction, were significant (*p* = 0.019 and *p* ≤ 0.0001, respectively) with respect to the bread shape coefficient (Table 6). A moderate positive correlation between the bread specific volume and shape coefficient was established (r = 0.439, *p* = 0.47). Significant differences between the control breads and breads prepared with 5% of Sc and with 15% of FSc mass loss after baking were not found. However, other bread sample groups showed 33.6% higher mass loss after baking. The interaction between analysed factors (scald fermentation and quantity interaction) was significant for bread mass loss after baking (*p* = 0.025) (Table 6).

As previously verified, the high temperature used during scalding affects starch gelatinization and protein denaturation, which results in greater flour viscosity and dough elasticity, and bread specific volume [40]. The increased specific volume of bread or millet cake made with heated flours has been reported [47,48]. However, gluten proteins may become denaturized as a result of scalding, and an excessively high amount of rye scald in the bread formula can diminish bread volume [49]. Thus, it was reported that scalded wholemeal could improve bread volume, but the addition of 10% of scalded flour in wheat bread resulted in a slightly lower bread volume [49]. Moreover, it was found that rye scald significantly affected the crumb cell diameter and area, as more cells per slice area could be obtained [40]. A lower pH in fermented rye scald may cause peptization and swelling of the proteins in the flour, increasing the consistency and subsequently the dough’s ability to retain carbon dioxide [50]. It has also been claimed that as the acidity rises, all of the moisture in the dough is bound by the undegraded starch, which may have an impact on mass loss after baking [51].

When comparing the colour characteristics of the bread crust, it was found that the addition of 10 and 15% of Sc, as well as 5, 10 and 15% of FSc, reduces bread crust lightness (L*), on average, by 18.2%. Analysed factors (scald fermentation and quantity) interaction were significant for bread crust L* (*p* = 0.0037) (Table 6). The lowest crust redness (a*) was obtained in breads prepared with 15% of Sc (in comparison with control breads and the group of breads prepared with FSc, on average, it was lower by 25.0%). Both analysed factors were significant on bread crust a* coordinates (scald fermentation *p* = 0.022 and scald quantity *p* = 0.048). Addition of Sc and FSc (except 5% of scalded flour) reduced bread crust yellowness (b*), in comparison with control samples, on average, by 14.0%. Scald quantity was a significant factor for bread crumb L* (*p* = 0.016) and the highest L* was recorded in samples prepared with 5 and 10% of Sc and with 10% of FSc (their L* coordinates were, on average, 57.2 NBS). The lowest crumb a* was found in the group of samples prepared with 15% of Sc (4.15 NBS). All the analysed factors and their interactions were significant for bread crumb a* (Table 6). The highest crumb b* was attained in the control group samples (19.9 NBS) and, by increasing the Sc the quantity in the main bread formula, crumb b* coordinates decreased (in samples prepared with 5% of Sc, on average, by 8.5%; in samples prepared with 10% of Sc, on average, by 19.1%; in samples prepared with 15% of Sc, on average, by 31.2%). Also, bread samples prepared with FSc showed lower b* coordinates in comparison with the control samples group (samples prepared with 5 and 15% of FSc, on average, lower by 24.1%; samples prepared with 10% of FSc, on average, lower by 20.6%). Scald quantity and scald fermentation x scald quantity interaction was significant for bread crumb b* coordinates (Table 6).

It has been reported that wheat bread with the addition of dietary fibre usually has a darker colour of crust and crumb [52]. The colour coordinates of the bread crust made with scalded rye in our study were similar with those obtained by Esteller et al. [53]. The higher percentage of seed coat and reducing sugars in rye wholemeal scald, as well as increased levels of some free amino acids in fermented scald, may contribute to the greater browning of baked bread [42,54]. This explains the lower values for the lightness of bread with Sc and FSc [53].

**Figure 2:**
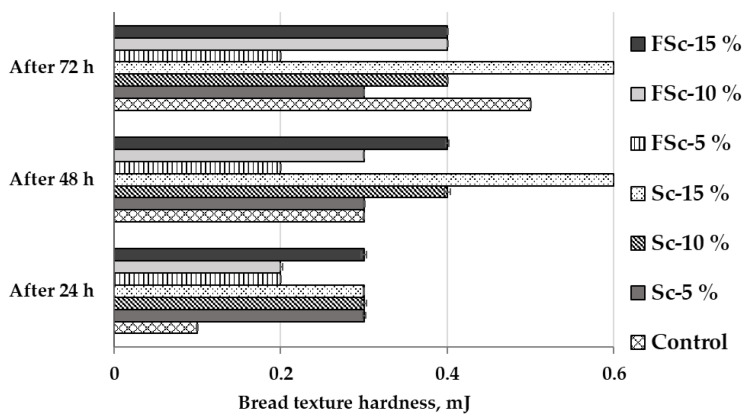
Bread texture hardness (mJ) after 24, 48 and 72 h of storage. Control—bread prepared without scald or fermented scald; Sc—scalded rye flour; FSc—scalded-fermented rye flour; 5%, 10% and 15%—bread prepared with 5%, 10% and 15%, respectively, scalded (Sc) or scalded-fermented (FSc) rye wholemeal flour.

Bread texture hardness after 24, 48 and 72 h of storage are shown in Figure 2. When comparing bread hardness after 24 h of storage, control bread hardness was the lowest (0.1 mJ). The hardness of bread prepared with Sc (5, 10 and 15%), as well as 15% of FSc was the highest, on average, at 0.3 mJ, and the hardness of bread prepared with 5 and 10% of FSc was, on average, 0.2 mJ. After 48 h of storage, different trends were established. Bread samples prepared with 5% of FSc showed lower hardness, in comparison with the control samples. After 72 h of storage, most of the bread groups prepared with Sc or FSc showed lower hardness in comparison with control breads (except for the bread group prepared with 15% of scalded flour).

Rye flour contains arabinoxylans, whose extractability and swelling properties increase under acidic conditions [55]. These compounds are essential in the water binding process and the formation of a viscous dough. Due to the effect of arabinoxylans and the decreased activity of amylase, rye bread made with sourdough hardens more slowly when compared with wheat bread [56]. Moreover, the prolonged shelf-life of bread prepared with sourdough may be related to the higher content of exopolysaccharides synthesized by LAB [32]. These effects may explain the reduced hardness of breads with Sc or FSc after 72 h of storage.

### 3.3. Sensory Properties and Overall Acceptability of Bread Samples

**Figure 3:**
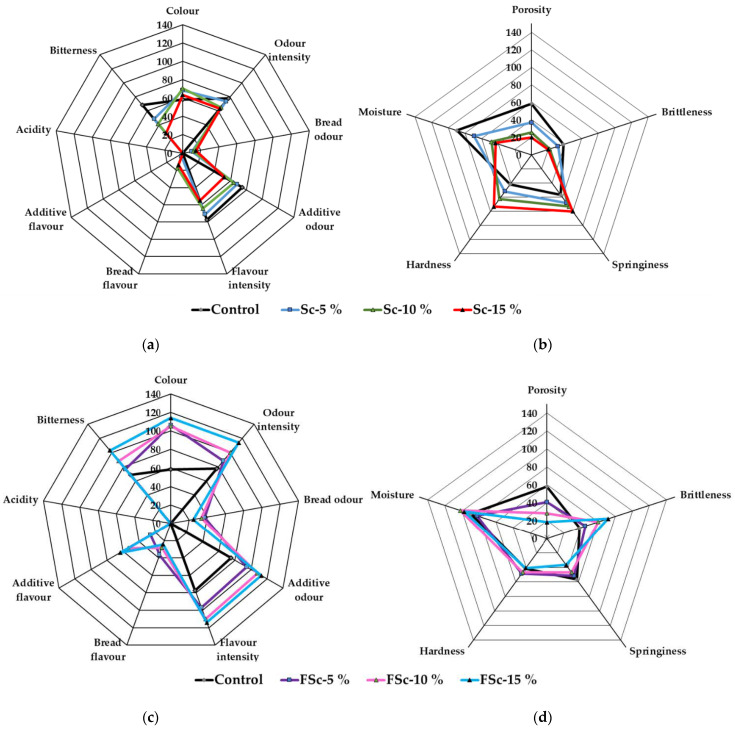

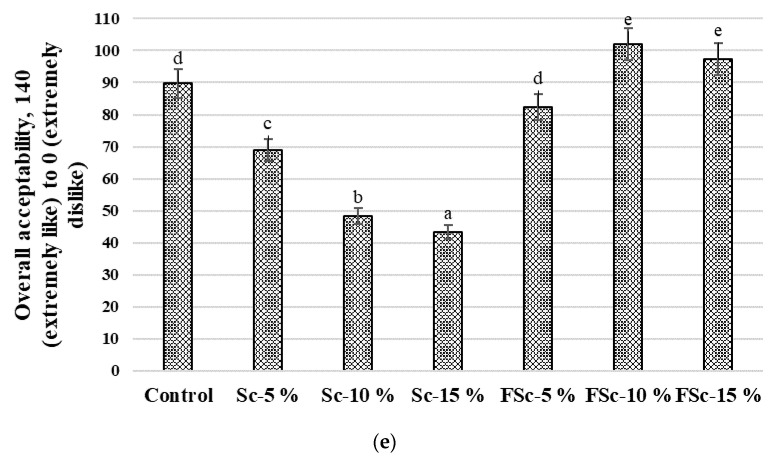
Bread sensory properties ((**a**,**c**)—colour, taste, flavour and odour sensory characteristics; (**b**,**d**)—texture sensory characteristics; and (**e**)—overall acceptability; control—bread prepared without scald or fermented scald; Sc—scalded rye flour; FSc—scalded fermented rye flour; 5%, 10%, 15%—bread prepared with 5%, 10%, 15%, respectively, scalded or scalded-fermented rye wholemeal flour. Data expressed as mean values (n = 10) ± standard error (SE). a–e Mean values within columns with different letters are statistically significantly different (*p* ≤ 0.05)).

Bread sensory properties are shown in Figure 3a,b—colour, taste, flavour and odour sensory characteristics; Figure 3c,d—texture sensory characteristics; and Figure 3e—overall acceptability. Comparing the colour sensory characteristics of bread, the bread sample groups prepared with FSc showed, on average, a 39.7% higher colour acceptability, in comparison with control samples and samples prepared with Sc. In addition, samples prepared with FSc showed a higher odour intensity (on average, 30.6% higher) in comparison with control samples and samples prepared with Sc. Addition of Sc to the main bread formula increased the bread odour intensity of the samples; in comparison with the Sc and FSc groups, on average a two times higher bread odour intensity was obtained in bread groups prepared with FSc. Bread sample groups prepared with FSc showed, on average, 0.6 times higher additive odour, in comparison with control samples and samples prepared with Sc. By increasing the percentage of Sc in the main bread formula, the flavour intensity of the samples was reduced; however, opposite tendencies were observed in bread prepared with FSc. The same tendencies were found in the bread flavour. The highest intensity of additive flavour was attained in bread group samples prepared with 10 and 15% of FSc (on average, 58.7). The acidity of all the tested bread groups was not sensorily felt, and the highest bitterness was exhibited in samples prepared with 15% of FSc. Analysis of between-subject effects showed that scald fermentation was a significant factor on all the analysed colour, taste, flavour and odour sensory characteristics (*p* ≤ 0.0001) (Table 7). However, scald quantity was a significant factor only for bread odour intensity and additive odour (*p* = 0.0003 and *p* ≤ 0.0001, respectively). Interaction of both analysed factors was significant for most of the analysed colour, taste, flavour and odour sensory characteristics (Table 7).

Regarding bread texture sensory characteristics (Figure 3b,d), the most acceptable porosity was evaluated in the control samples and breads prepared with 5% of FSc. All the bread groups prepared with FSc showed, on average, 2.1 times higher brittleness, in comparison with control samples and breads prepared with Sc. Significant differences in the springiness and the texture moisture between the bread groups were not found. Nevertheless, the hardest samples were obtained in the bread groups prepared with 15% Sc. Analysed factors and their interaction were significant on most of the analysed bread texture sensory characteristics except in scald fermentation. However, the interaction of factors was not significant for bread porosity, and scald quantity was not significant for bread springiness.

When comparing bread overall acceptability, significantly higher overall acceptability was exhibited in bread groups prepared with FSc, in comparison with control group breads and breads prepared with Sc (Figure 3e). Furthermore, scald fermentation and interaction of scald quantity and fermentation were significant for bread overall acceptability (Table 7).

Bread flavour is highly affected by the lactic and acetic acids, free amino acids, exopolysaccharides and volatile compounds (alcohols, aldehydes, ethers, etc.) formed during sourdough fermentation [57]. Different sourdoughs have shown to improve the sensory qualities of wheat breads [58]. Moreover, heat treatment of flour causes variations in flavour and browning during baking. Gao et al. (2018) observed higher sensory scores of rye-wheat breads made with heat-treated rye flour [40]. Djukic et al. (2013) reported that with the addition of rye scald to bread dough, the sensory qualities of the rye/wheat bread were enhanced [50]. Higher scores of bitterness for breads with Sc and FSc could be explained by the presence of wholemeal flour, which contains bitter taste-eliciting bran [59].

### 3.4. Acrylamide Concentration in Bread

**Figure 4:**
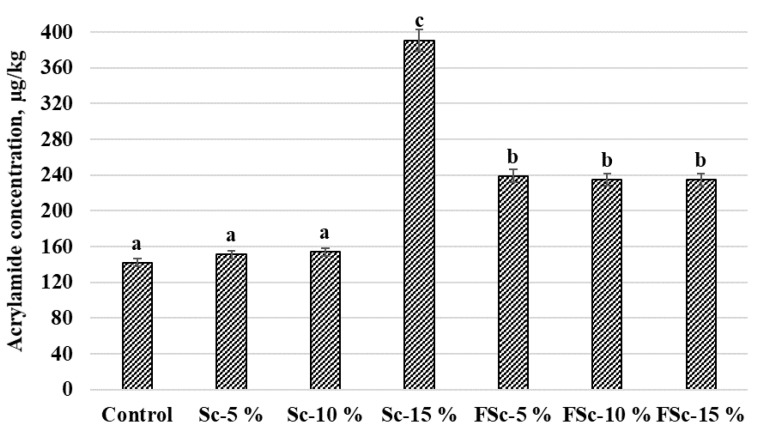
Acrylamide concentration (µg/kg) in bread samples (a–c Mean values within columns with different letters are statistically significantly different (*p* ≤ 0.05)).

Acrylamide concentrations (µg/kg) in the bread samples are given in Figure 4. The lowest concentration of acrylamide was found in the group of control samples and breads prepared with 5 and 10% of Sc (on average, 149.0 µg/kg). Acrylamide content in samples prepared with FSc was, on average, 236.3 µg/kg. However, samples prepared with 15% of Sc had, on average, a 61.9% higher acrylamide concentration compared with control samples and samples prepared with 5 and 10% of Sc, and, on average, a 39.6% higher acrylamide concentration compared with the groups of samples prepared with FSc. Moderate negative correlations between acrylamide concentration in bread and bread shape coefficient, bread crumb L* and b* colour coordinates were obtained (r = −0.528, *p* = 0.014; r = −0.680, *p* ≤ 0.001; r = −0.443, *p* = 0.044, respectively). Scald quantity and scald fermentation and quantity interaction were significant for acrylamide concentration in bread samples (Table 8).

The potentially carcinogenic acrylamide is produced during the Maillard reaction and its main precursors are reducing sugars such as glucose and fructose, and the amino acid asparagine, which is a limiting precursor [60]. In addition to its high nutritional value, rye flour also contains a higher content of asparagine than wheat [16]. It has been reported that greater acrylamide formation in rye bread can also occur due to the high content of dietary fibre and ash in flour [61]. However, fermentation with LAB can lead to a reduction in acrylamide content in bread because the LAB use free asparagine in their metabolism [60]. Przygodzka et al. (2015) reported a weak effect of wheat flour extraction rates and their chemical components on acrylamide formation in breads baked at 240 °C, while this effect was not found in rye flour [16]. It was also claimed that the level of acrylamide was lower when bread was baked at a lower temperature with a longer baking time. In our study, the content of asparagine and fructose were similar in Sc and FSc; only glucose content was higher in FSc compared with Sc, and that probably led to the higher content of acrylamide in breads with FSc. This could also suggest that the starter culture did not successfully lower the free asparaginase content.

## 4. Conclusions

The addition of various cereal varieties to wheat bread could lead to healthier, but still tasty, products. The preparation of scalded rye flour is very common technology for rye bread production in Eastern Europe. It is valuable owing to its ability to increase the sweet taste of bread without addition of saccharose—a positive influence on the glycaemic index—while fermentation of scalded flour leads to a desirable sweet–sour bread taste and the extension of bread shelf-life. As studies about the rye scald fermentation with certain LAB and further uses for semi-wheat-rye bread production are still scarce, our study provides beneficial information in this field. We used the newly isolated *Lactiplantibacillus paracasei* No. 244 strain—which possesses versatile carbohydrate metabolism, tolerance to acidic conditions, and antimicrobial properties—for the rye scald fermentation. The outcome of this study showed that scalding caused higher levels of reducing sugars in rye wholemeal. Fermentation decreased the pH and hardness but increased the TTA and redness (a*) value of scald. In most cases, amino acid concentrations in Sc and FSc were lower than in rye wholemeal flour. However, fermentation of Sc increased the levels of certain amino acids, as well as GABA.

Scald type (unfermented and fermented) and quantity (5, 10 and 15%) affected the quality of semi-wheat-rye bread in certain parameters. Colour coordinates, shape coefficient and mass loss after baking of semi-wheat-rye breads were significantly influenced by the quantity and type of scald. Reduced hardness of breads with Sc (except with 15% of Sc) or FSc was observed after 72 h of storage, compared with control samples. Scald fermentation was a significant factor on such sensory characteristics as colour, taste, flavour and odour of bread. Addition of FSc significantly improved overall acceptability of the tested bread. Compared with control bread, addition of 5 and 10% of scald to the bread formula did not enhance the formation of acrylamide in wheat-rye bread. In summary, scald fermented with *Lp. paracasei* No. 244 enriches bread with GABA and improves bread sensory acceptability as well as staling.

## Figures and Tables

**Table 1:** Characteristics of the *Lactiplantibacillus paracasei* No. 244 strain.

100 bp DNA-Ladder Extended	*Lactiplantibacillus paracasei* No. 244	Gas Production, Tolerance to Temperature (10, 30, 37 and 45 °C), Low pH Conditions (pH 2.5 for 2 h) and Carbohydrate Metabolism of the *Lactiplantibacillus paracasei* No. 244 Strain			
** 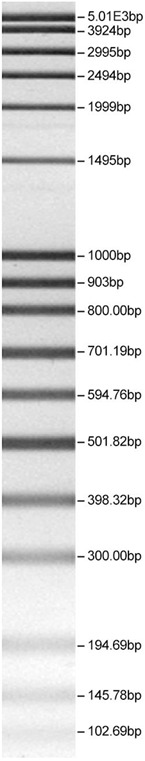 **	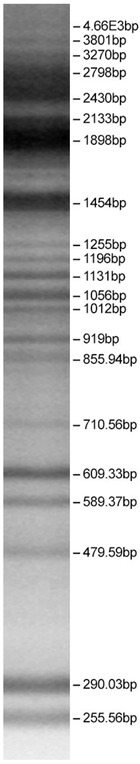	Glycerol	-	Esculin	+++
		D-arabinose	-	Salicin	+++
		L-arabinose D-ribose D-xylose L-xylose D-adonitol Methyl-βD-xylopiranoside D-galactose D-glucose D-fructose D-mannose L-sorbose L-rhamnose Dulcitol Inositol D-mannitol D-sorbitol Methyl-αD-mannopyranoside Methyl-αD-glucopyranoside N-acetylglucosamine Amygdalin Arbutin	- +++ - +++ + - +++ +++ +++ +++ - +++ +++ - +++ +++ - +++ +++ +++ +++	D-cellobiose D-maltose D-lactose D-melibiose D-saccharose D-trehalose Inulin D-melezitose D-raffinose Amidon Glycogen Xylitol Gentiobiose D-turanose D-tagatose D-fucose L-fucose D-arabitol L-arabitol Potassium gluconate Potassium 2-ketogluconate Potassium 5-ketogluconate	+++ +++ +++ - +++ +++ +++ +++ - - - - +++ +++ +++ - - - - ++ - -
		Gas production (+/-)		-	
		Temperature tolerance		10 °C	-
				30 °C	++
				37 °C	++
				45 °C	-
		pH 2.5		0 h log (CFU/mL)	9.41 ± 0.2
				2 h log (CFU/mL)	9.29 ± 0.1

CFU—colony forming units; interpretation of lactic acid bacteria (LAB) growth in API 50 CH system and API 20 E system: +++ = strong growth (yellow); ++ = moderate growth (green); + = weak growth (dark green); - = no growth (blue).

**Table 2:** Acidity characteristics (pH and total titratable acidity), colour coordinates and hardness of scalded (Sc) and scalded-fermented (FSc) (after 0 and 24 h of fermentation) rye wholemeal flour.

Samples	Acidity Parameters		Colour Characteristics, NBS			Texture Hardness, mJ
	pH	TTA, °N	L*	a*	b*	
Sc	6.22 ± 0.06 c	1.04 ± 0.46 a	35.13 ± 4.31 a	2.46 ±0.25 a	8.80 ± 1.27 a	0.700 ± 0.100 b
FSc-0h	5.95 ± 0.13 b	1.23 ± 0.16 a	34.37 ± 4.25 a	2.56 ± 0.20 a	10.8 ± 1.10 b	0.770 ± 0.100 b
FSc-24h	4.57 ± 0.05 a	2.53 ± 0.15 b	32.29 ± 0.08 a	2.80 ± 0.21 b	7.49 ± 0.01 a	0.200 ± 0.050 a

TTA—total titratable acidity; L* lightness; a* redness or −a* greenness; b* yellowness or −b* blueness; NBS—National Bureau of Standards units. Sc—scalded rye wholemeal flour; FSc—scalded-fermented rye flour; 0h—before fermentation; 24h—after 24 h of fermentation. Data expressed as mean values (n = 3) ± standard error (SE). a–c Mean values within a row with different letters are statistically significantly different (*p* ≤ 0.05).

**Table 3:** Sugars (fructose, glucose, sucrose, maltose) concentration in rye wholemeal flour, and scalded (Sc) and scalded-fermented (FSc) (after 24 h) rye wholemeal samples.

Samples	Sugars, g/100 g			
	Monosaccharides		Disaccharides	
	Fructose	Glucose	Sucrose	Maltose
Rye wholemeal flour	nd	nd	1.61 ± 0.09	1.02 ± 0.09 a
Sc	0.880 ± 0.034 a	1.25 ± 0.08 a	nd	6.26 ± 0.14 b
FSc-24h	0.820 ± 0.053 a	1.44 ± 0.03 b	nd	6.95 ± 0.23 b

Sc—scalded rye wholemeal flour; FSc—scalded-fermented rye flour; 24h—after 24 h of fermentation; nd—not determined. Data expressed as mean values (n = 3) ± standard error (SE). a,b Mean values within a row with different letters are statistically significantly different (*p* ≤ 0.05).

**Table 4:** Free amino acid profile and gamma aminobutyric acid (GABA) concentration in rye wholemeal flour and in scalded (Sc) and scalded-fermented (FSc) (after 24 h) rye wholemeal samples.

Compound Name	Rye Wholemeal Flour	Sc	FSc-24h
	Free Amino Acids and GABA Concentration, µmol/g		
Asparagine	5.19 ± 0.26 b	2.32 ± 0.17 a	2.11 ± 0.16 a
Arginine	1.94 ± 0.12 c	0.855 ± 0.041 a	1.08 ± 0.09 b
Serine	0.785 ± 0.031 b	0.368 ± 0.025 a	0.354 ± 0.028 a
Aspartic acid	2.33 ± 0.09 b	0.528 ± 0.031 a	0.517 ± 0.036 a
Glutamic acid	0.896 ± 0.039 c	0.281 ± 0.019 a	0.321 ± 0.021 b
Threonine	0.516 ± 0.028 c	0.173 ± 0.011 a	0.308 ± 0.018 b
Glycine	0.453 ± 0.015 b	0.259 ± 0.022 a	0.429 ± 0.022 b
GABA	0.456 ± 0.021 a	0.482 ± 0.034 a	0.689 ± 0.043 b
Alanine	1.56 ± 0.11 c	0.684 ± 0.041 a	1.17 ± 0.08 b
Proline	2.24 ± 0.15 b	0.967 ± 0.053 a	0.968 ± 0.039 a
Methionine	nd	nd	nd
Valine	nd	nd	nd
Phenylalanine	nd	nd	nd
Lysine	nd	nd	nd
Histidine	nd	nd	nd
Tyrosine	nd	nd	nd

Sc—scalded rye wholemeal flour; FSc—scalded-fermented rye flour; 24h—after 24 h of fermentation; GABA—gamma aminobutyric acid; nd—not determined. Data expressed as mean values (n = 3) ± standard error (SE). a–c Mean values within a row with different letters are statistically significantly different (*p* ≤ 0.05).

**Table 5:** Bread specific volume, porosity, shape coefficient, mass loss after baking, colour characteristics of the bread crust and crumb, and bread crumb images.

Bread Samples	Specific Volume, cm^3^/g				Shape Coefficient					Mass Loss after Baking, %	
Control	1.90 ± 0.11 a				2.38 ± 0.19 b					6.30 ± 0.83 a	
Sc-5%	2.09 ± 0.09 a				2.64 ± 0.22 b					6.30 ± 0.83 a	
Sc-10%	2.00 ± 0.12 a				2.67 ± 0.19 b					11.2 ± 0.86 b	
Sc-15%	2.01 ± 0.08 a				1.89 ± 0.10 a					11.0 ± 3.28 b	
FSc-5%	2.06 ± 0.16 a				2.56 ± 0.14 b					12.0 ± 0.55 b	
FSc-10%	1.87 ± 0.05 a				2.60 ± 0.36 b					11.4 ± 0.68 b	
FSc-15%	1.94 ± 0.17 a				1.70 ± 0.35 a					9.25 ± 4.37 a,b	
**Bread samples**	**Crust**						**Crumb**				
	L*		a*	b*			L*		a*		b*
Control	50.7 ± 4.14 b		8.62 ± 0.54 b	17.4 ± 1.55 b			53.0 ± 3.91 a,b		6.65 ± 0.28 d		19.9 ± 0.57 f
Sc-5%	52.3 ± 4.57 b		7.44 ± 0.84 a,b	16.2 ± 1.01 b			58.7 ± 4.39 b		6.98 ± 0.20 d		18.2 ± 0.58 e
Sc-10%	43.7 ± 2.68 a		8.26 ± 1.41 a,b	14.6 ± 2.13 a			58.3 ± 5.75 b		4.63 ± 0.48 b,c		16.1 ± 0.24 d
Sc-15%	40.1 ± 3.85 a		6.50 ± 1.49 a	13.8 ± 3.87 a			48.3 ± 1.27 a		4.15 ± 0.06 a		13.7 ± 0.36 a
FSc-5%	40.8 ± 2.44 a		8.36 ± 0.39 b	13.6 ± 0.90 a			51.5 ± 1.63 a		4.71 ± 0.06 b		15.1 ± 0.36 b
FSc-10%	44.3 ± 2.45 a		9.38 ± 0.62 b	15.1 ± 1.12 a,b			54.7 ± 0.19 b		4.89 ± 0.07 c		15.8 ± 0.19 c
FSc-15%	41.7 ± 3.64 a		8.03 ± 0.99 b	13.1 ± 2.43 a			51.3 ± 1.15 a		4.83 ± 0.16 a,b		15.1 ± 0.41 b
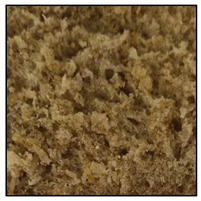			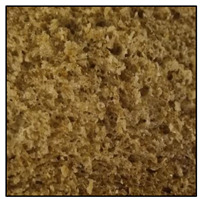					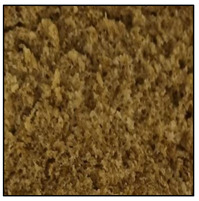			
Control			Sc-5%					Sc-10%			
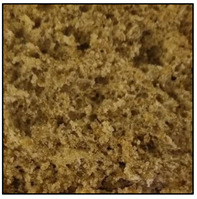		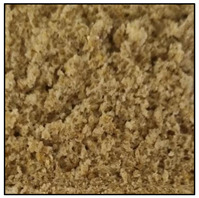				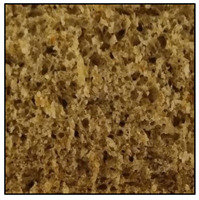				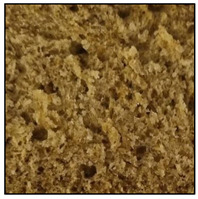	
Sc-15%		FSc-5%				FSc-10%				FSc-15%	

Data expressed as mean values (n = 3) ± standard error (SE). a–f Mean values within a row with different letters are statistically significantly different (*p* ≤ 0.05). L* lightness; a* redness or −a* greenness; b* yellowness or −b* blueness; NBS—National Bureau of Standards units. Control—bread prepared without scald or fermented scald; Sc—scalded rye flour; FSc—scalded-fermented rye flour; 5, 10 and 15%—bread prepared with 5, 10 and 15%, respectively, scalded or scalded-fermented rye wholemeal flour.

**Table 6:** Influence of analysed factors (fermentation and quantity of the scald, and their interaction) on bread specific volume, porosity, shape coefficient, mass loss after baking, and crust and crumb colour coordinates.

Bread Parameters		Factors and Their Interaction		
		Scald Fermentation	Scald Quantity	Scald Fermentation and Quantity Interaction
Specific volume, cm^3^ g^−1^		0.188	0.139	0.785
Shape coefficient		0.321	0.019	0.0001
Mass loss after baking, %		0.205	0.259	0.025
Crust	L*	0.153	0.111	0.037
	a*	0.022	0.048	0.863
	b*	0.374	0.416	0.458
Crumb	L*	0.154	0.016	0.080
	a*	0.004	0.0001	0.0001
	b*	0.240	0.014	0.014

Influence of analysed factors (fermentation and quantity of the scald) on bread parameters is statistically significant when *p* ≤ 0.05.

**Table 7:** Influence of analysed factors (fermentation and quantity of the scald, and their interaction) on bread sensory properties and overall acceptability.

Bread Parameters	Factors and Their Interaction		
	Scald Fermentation	Scald Quantity	Scald Fermentation and Quantity Interaction
Colour	0.0001	0.953	0.189
Odour intensity	0.0001	0.003	0.0001
Bread odour	0.0001	0.322	0.012
Additive odour	0.0001	0.413	0.002
Flavour intensity	0.0001	0.424	0.0001
Bread flavour	0.0001	0.455	0.004
Additive flavour	0.0001	0.0001	0.0001
Acidity	-	-	-
Bitterness	0.0001	0.593	0.0001
Porosity	0.405	0.0001	0.508
Brittleness	0.0001	0.007	0.0001
Springiness	0.0001	0.685	0.0001
Hardness	0.0001	0.006	0.0001
Moisture	0.0001	0.003	0.0001
Overall acceptability	0.0001	0.395	0.0001

- not detected. Influence of analysed factors (fermentation and quantity of the scald) on bread parameters is statistically significant when *p* ≤ 0.05.

**Table 8:** Influence of analysed factors (fermentation and quantity of the scald, and their interaction) on acrylamide concentration in bread.

Bread Parameters	Factors and Their Interaction		
	Scald Fermentation	Scald Quantity	Scald Fermentation and Quantity Interaction
Acrylamide concentration	0.172	0.0001	0.0001

Influence of analysed factors (fermentation and quantity of the scald) on bread parameters is statistically significant when *p* ≤ 0.05.

## Data Availability

The data are available from the corresponding author, upon reasonable request.
